# Factors Associated With Professional Socialization Among Korean Male Nurses: A Cross‐Sectional Study

**DOI:** 10.1155/jonm/1530540

**Published:** 2026-02-19

**Authors:** Jae Jun Lee, Yeonsoo Jang, Soo Young Han, You Lee Yang, Young Man Kim, Eui Geum Oh

**Affiliations:** ^1^ Mo-Im KIM Nursing Research Institute, College of Nursing, Yonsei University, Seoul, 03722, Republic of Korea, yonsei.ac.kr; ^2^ Division of Nursing, Severance Hospital, Seoul, 03722, Republic of Korea, yuhs.or.kr; ^3^ College of Nursing, Eulji University, Seongnam, 13135, Republic of Korea, eulji.ac.kr; ^4^ Red Cross College of Nursing, Chung-Ang University, Seoul, 06794, Republic of Korea, cau.ac.kr

**Keywords:** gender role, male, nurses, self-concept, self-efficacy, social support, socialization, working conditions

## Abstract

**Background:**

Although the number of male nurses is steadily increasing in South Korea, nursing remains a female‐dominated profession, and male nurses continue to face unique social and organizational challenges. Professional socialization plays a critical role in their professional development, yet little is known about the factors that influence this process among male nurses.

**Objective:**

To identify individual, interpersonal, and organizational factors associated with professional socialization among Korean male nurses.

**Design:**

A descriptive correlational, cross‐sectional study.

**Methods:**

Data from 194 male nurses working in hospitals were analyzed through an online survey conducted between June and September 2023. The independent variables included four individual factors (e.g., self‐efficacy, professional self‐concept, gender role conflict, and clinical experience), one interpersonal factor (e.g., social support), and one organizational factor (e.g., nursing work environment). Hierarchical linear regression was used to identify factors associated with professional socialization.

**Results:**

In the final hierarchical linear regression model (adjusted R^2^ = 0.705), self‐efficacy, professional self‐concept, social support, and a more favorable nursing work environment were independently associated with higher professional socialization, whereas gender role conflict and clinical experience were not significant predictors.

**Conclusion:**

This study highlights the multidimensional nature of professional socialization among male nurses, emphasizing the need for coordinated efforts at individual, interpersonal, and organizational levels.

**Implication for Nursing Management:**

Nurse managers and healthcare organizations should consider targeted strategies (such as mentorship programs, peer support networks, and inclusive workplace policies) to enhance professional socialization among male nurses.

## 1. Introduction

The number of men entering the nursing profession has steadily increased worldwide, driven by high employment rates, job security, and growing societal recognition of nursing as a legitimate profession [1]. In South Korea, the proportion of male nurses in healthcare settings rose from 1.5% in 2010 to 5.1% in 2020, marking nearly a threefold increase over a decade [2]. Furthermore, as of 2024, 25.0% of first‐year nursing students were male, suggesting a continued upward trend [3].

Despite this progress, nursing continues to be perceived as a female‐dominated profession, and men entering the field may still face social stigma related to gender role stereotypes or assumptions about their identity [4]. Additionally, male nurses experience rejection from patients or caregivers [5] and struggle to build relationships and communicate within nursing organizations, which are primarily composed of women [6]. These challenges contribute to job dissatisfaction and may discourage long‐term commitment to the profession [5].

Professional socialization plays a crucial role in nurses’ professional development, influencing their decision to stay in or leave the profession [7]. Professional socialization is the process through which individuals acquire the values, skills, knowledge, behaviors, and norms necessary to fulfill their purposes within a profession that are essential for transitioning to professional roles [8]. This process occurs through the integration of nursing‐specific knowledge and skills learned through lectures or education with clinical experience, from nursing school to retirement in the profession [9]. Successful socialization improves job satisfaction, enhances the quality of nursing care, and reduces turnover, highlighting the importance of expediting this process [10].

As professional socialization is a complex and ongoing process, it is important to be aware of the factors influencing it to promote positive outcomes [8]. Mariet [11] conducted a literature review to synthesize various conceptual models of professional socialization in nursing. This review identified several key factors that contribute to professional socialization, including self‐confidence, personal beliefs, and clinical experiences. Salisu et al. [9] conducted a systematic review to identify the factors that promote or hinder professional socialization in nursing, categorizing them into three main themes: professional, personal, and educational. Professional factors included theory–practice incongruence, professional support, and workplace relationships; personal factors encompassed dependence, individual beliefs, and learning motivation; and educational factors comprised greater education, supportive mentors, and interactive learning support. However, there is still a lack of research identifying the factors associated with socialization among nurses.

Furthermore, existing research on professional socialization in nursing has focused on female nurses or the overall nursing population, where females dominate, with limited attention specifically paid to male nurses. Empirical research that directly examines male nurses’ professional socialization is scarce, with only two studies [7, 12] reported to date. LaRocco [12] studied 28 male American nurses and reported that family and social support, perceptions of fellow nurses, and experiences of discrimination impacted professional socialization. Azadi et al. [7] examined 22 male Iranian nurses and highlighted that the perception of nursing as a female‐dominated occupation, lack of support from family and peers, and unfavorable work environments hinder professional socialization. Notably, both studies highlight the significance of support from family and peers in coping with gender‐based inequalities. This underscores the necessity to consider not only individual factors but also interpersonal and organizational factors for male nurses’ professional socialization. However, both studies employed qualitative approaches, and to date, there is a lack of quantitative evidence on the factors affecting professional socialization among male nurses. Understanding the factors associated with professional socialization among male nurses can inform interventions and support systems to facilitate the smoother integration of men into the nursing workforce. Therefore, this study aimed to identify factors associated with professional socialization among male nurses.

This study’s theoretical framework was based on Bronfenbrenner’s ecological systems theory [13]. The Bronfenbrenner’s model categorizes environments that impact individuals, both directly and indirectly, from the closest to the farthest, emphasizing the dynamic interactions between individuals and their environments to explain human development and growth [13]. This ecological perspective has the advantage of allowing for a multifaceted understanding and approach to humans by identifying the influential factors for each aspect.

**FIGURE 1:**
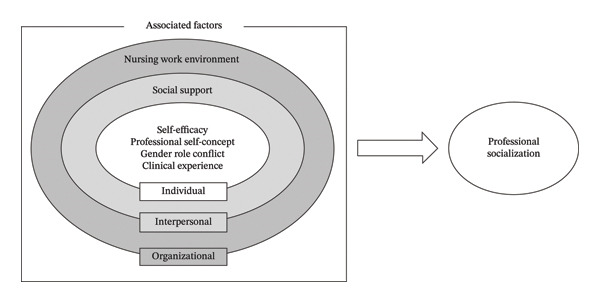
Conceptual framework of professional socialization in male nurses.

Accordingly, this study conceptualizes the factors influencing professional socialization across three levels: individual, interpersonal, and organizational. Self‐efficacy, professional self‐concept, gender role conflict, and clinical experience reflect individual perceptions, behaviors, and accumulated practical expertise [14] and were therefore included at the individual level. Social support, including the support and assistance received from others, is included at the interpersonal level. The nursing work environment, encompassing interactions between the organization and nurses, is included at the organizational level (Figure 1). Professional socialization among male nurses is thus viewed as emerging from dynamic interactions between individual attributes, interpersonal relationships, and the organizational context.

## 2. Materials and Methods

### 2.1. Study Design

This study employed a descriptive correlational, cross‐sectional design.

### 2.2. Sample and Data Collection

Male nurses working in hospitals, who understood the purpose and intention of our study, were invited to participate. Nurses who were on leave at the time of data collection were excluded. The G‐Power 3.1 program was used to determine the appropriate sample size for the regression analysis. Assuming a Type I error of 0.05, a statistical power of 90%, 20 independent variables, and a medium effect size of 0.15 [15], the required sample size was calculated to be 191. Accounting for a dropout rate of 10%, the targeted recruitment was 213 participants.

Data were collected using an online survey between June and September 2023. Quota sampling was applied based on the regional distribution of male nurses, referencing the 2021 healthcare workforce survey in South Korea [2]. The purpose and procedures of the study were explained by telephone to hospitals nationwide, and 16 institutions agreed to participate. Nursing departments of these hospitals supported recruitment by distributing the survey invitation email containing a link to the online questionnaire.

A total of 217 participants responded to the online survey. To enhance data quality, the survey system was configured to allow only one submission per device. Among these respondents, 12 individuals who were not currently employed were excluded. We then conducted data screening and excluded 11 responses due to potential duplicate entries or implausibly rapid completion (< 120 s). This completion‐time criterion was used as a pragmatic data‐quality check, as prior web‐based survey methodology has applied similar minimum time thresholds to flag potentially inattentive or patterned responding; accordingly, we used a conservative cutoff of 120 s to reduce the likelihood of low‐quality responses [16]. Consequently, data from 194 participants (89.4%) were included in the final analysis.

### 2.3. Ethical Considerations

This study was approved by the Institutional Review Board of Yonsei University Health System (IRB No: 4‐2023‐0508). The online survey introduction page provided comprehensive information about the study, including its purpose, inclusion, and exclusion criteria, and estimated completion time. Participation was voluntary, and respondents could withdraw at any time without penalty. Informed consent was obtained electronically by requiring participants to check mandatory boxes at the beginning of the survey to confirm their understanding and agreement to participate. As a token of appreciation, we offered a gift card worth approximately US$5 to each participant.

### 2.4. Measurements

All instruments were administered in Korean‐validated versions. Where an instrument was originally developed in English, we used a validated Korean translation with published psychometric evidence; where the instrument was originally developed in Korea, we used the original Korean tool.

#### 2.4.1. Professional Socialization

Professional socialization was measured using a professional socialization scale, which was developed and validated among hospital nurses in Korea [17]. This scale comprises 21 items across four domains: ethical practice and reflection (eight items), perception of respect and recognition (three items), clinical competency based on leadership (five items), and desires and motivation for professional development (five items). These items were scored on a 6‐point Likert scale ranging from 1 (*strongly disagree*) to 6 (*strongly agree*). The total scores ranged from 21 to 126, with higher values indicating higher professional socialization. In the instrument development study, the overall Cronbach’s alpha was 0.95, and the values for the four domains were 0.90, 0.84, 0.88, and 0.89, respectively [17]. In this study, the overall Cronbach’s alpha was 0.92, with domain‐specific values of 0.77, 0.83, 0.78, and 0.84, respectively.

#### 2.4.2. Self‐Efficacy

The Nursing Self‐Efficacy Scale was used to measure self‐efficacy [18]. This tool was modified and supplemented by Park et al. [18] for use with Korean nurses to measure self‐efficacy in nursing situations, based on the generalized expectations of the self‐efficacy tool developed by Sherer and Adams [19]. This tool consists of seven items that use a 4‐point Likert scale ranging from 1 (*strongly disagree*) to 4 (*strongly agree*), with higher scores indicating greater self‐efficacy. At the time of development, the reliability was Cronbach’s alpha 0.88 [18], and it was 0.76 in this study.

#### 2.4.3. Professional Self‐Concept

Professional self‐concept was measured using the Korean version of the 27‐item Professional Self‐concept Instrument for Nurses [20]. Each item was scored on a 4‐point Likert scale ranging from 1 (*disagree*) to 4 (*agree*), with higher scores indicating higher levels of professional self‐concept. The instrument showed a Cronbach’s alpha of 0.85 in prior research [20] and 0.75 in this study.

#### 2.4.4. Gender Role Conflict

Gender role conflict was measured using the 27‐item Korean Male Gender Role Conflict Scale [21], developed to measure the characteristics of gender role conflicts in Korean men. Each item is measured on a 6‐point Likert scale ranging from 1 (*strongly disagree*) to 6 (*strongly agree*), with higher scores indicating severe gender role conflict. The Cronbach’s alpha was 0.90 in the previous study [21] and 0.96 in this study.

#### 2.4.5. Clinical Experience

Clinical experience was measured using a self‐reported item asking how long participants had worked in their current unit.

#### 2.4.6. Social Support

The Korean version of the 10‐item Male Nurses’ Social Support Scale was used to measure social support [22]. Each item is measured on a 5‐point scale ranging from 1 (*strongly disagree*) to 5 (*strongly agree*), with higher scores indicating greater social support. Cronbach’s alpha was 0.85 in a previous study [22] and 0.84 in this study.

#### 2.4.7. Nursing Work Environment

Nursing work environment was measured using the 29‐item Korean version of the Practice Environment Scale of the Nursing Work Index (PES‐NWI) [23]. Each item was measured on a 4‐point scale ranging from 1 (*strongly disagree*) to 4 (*strongly agree*), with higher scores indicating a better nursing work environment. Cronbach’s alpha for all items was 0.93 in a previous study [23] and 0.95 in this study.

#### 2.4.8. General and Occupational Characteristics

General and occupational characteristics were measured based on previous studies [17, 24]. General characteristics included age, educational level, and marital status. Occupational characteristics included the type of hospital, gender of the preceptor, existence of male nurse colleague in the unit, assignment to the desired unit, residential area, and working unit. Residential areas were categorized as capital area (Seoul, Gyeonggi, and Incheon) and noncapital area (all other regions) based on the Korean capital region classification.

### 2.5. Data Analysis

Descriptive statistics were presented as mean and standard deviation (SD). Pearson’s correlation coefficient was used to analyze the correlations between professional socialization, self‐efficacy, professional self‐concept, gender role conflict, clinical experience, social support, and the nursing work environment. Hierarchical linear regression analysis was used to identify the factors associated with professional socialization among male nurses. All statistical analyses were performed using SAS (Version 9.4; SAS Institute Inc.), and the statistical significance level was set at *p* < 0.05.

## 3. Results

### 3.1. General and Occupational Characteristics of Participants

As shown in Table 1, the mean age was 31.88 ± 3.70 years. Of the participants, 5.2% had a master’s or doctoral degree, and 79.4% were not married, 11.3% were male preceptors, and nearly half (45.9%) had male nurse colleagues in the unit.

**TABLE 1:** General and occupational characteristics of participants (*N* = 194).

Variable	Category	Mean ± SD or *N* (%)
Age (years)		31.88 ± 3.70

Educational level	Bachelor	184 (94.8)
	≥ Master	10 (5.2)

Marital status	Married	39 (20.1)
	Not married	154 (79.4)
	Bereaved/divorced	1 (0.5)

Type of hospital	Tertiary hospital	76 (39.2)
	General hospital	109 (56.2)
	Hospital	9 (4.6)

Gender of preceptor	Male	22 (11.3)
	Female	172 (88.7)

Existence of male nurse colleague in the unit	Yes	89 (45.9)
	No	105 (54.1)

Assignment to the desired unit	Yes	147 (75.8)
	No	47 (24.2)

Residential area	Capital	90 (46.4)
	Non‐capital	104 (53.6)

Working unit	General ward	93 (47.9)
	Operating/intervention room	24 (12.4)
	Emergency department	22 (11.3)
	Laboratory room	16 (8.3)
	Outpatient department	15 (7.7)
	Anesthetic/recovery room	15 (7.7)
	Intensive care unit	9 (4.6)

### 3.2. Descriptive Statistics for Study Variables

The mean self‐efficacy score was 23.12 ± 2.66 (possible range 7–28). The mean professional self‐concept score was 76.69 ± 7.09 (range 27–108), and the mean gender role conflict score was 110.04 ± 30.24 (range 27–162). Participants had an average of 3.05 ± 2.46 years of clinical experience in their current unit. The mean scores for social support and nursing work environment were 40.40 ± 4.80 (range 10–50) and 87.39 ± 12.99 (range 29–116), respectively (Table 2).

**TABLE 2:** Descriptive statistics for study variables (*N* = 194).

Study variable	Mean ± SD	Range
Self‐efficacy	23.12 ± 2.66	7–28
Professional self‐concept	76.69 ± 7.09	27–108
Gender role conflict	110.04 ± 30.24	27–162
Clinical experience (years)	3.05 ± 2.46	
Social support	40.40 ± 4.80	10–50
Nursing work environment	87.39 ± 12.99	29–116

### 3.3. Professional Socialization and Subfactor Scores

The mean score for professional socialization was 4.71 ± 0.56 out of 6 points, indicating a moderate‐to‐high level. Among the four subfactors, ethical practice and reflection was the highest, with a mean score of 4.88 ± 0.49, followed by desires and motivation for professional development (4.76 ± 0.70), clinical competency based on leadership (4.58 ± 0.67), and perception of respect and recognition (4.41 ± 0.93) (Table 3).

**TABLE 3:** Professional socialization and subfactor scores (*N* = 194).

Variable	Number of items	Range	Mean ± SD	Rank
Professional socialization (total)	21	1–6	4.71 ± 0.56	—
Ethical practice and reflection	8	1–6	4.88 ± 0.49	1
Perception of respect and recognition	3	1–6	4.41 ± 0.93	4
Clinical competency based on leadership	5	1–6	4.58 ± 0.67	3
Desires and motivation for professional development	5	1–6	4.76 ± 0.70	2

*Note:* Rank was assigned by ordering subfactor mean scores from highest (1) to lowest (4).

### 3.4. Correlation Among Professional Socialization and Study Variables

Professional socialization was positively correlated with self‐efficacy, professional self‐concept, social support, and nursing work environment, whereas it was negatively correlated with gender role conflict and showed no significant correlation with clinical experience (Table 4).

**TABLE 4:** Correlation among professional socialization and study variables (*N* = 194).

Variable	r (*p*)						
	Professional socialization	Self‐efficacy	Professional self‐concept	Gender role conflict	Clinical experience	Social support	Nursing work environment
Professional socialization	1						
Self‐efficacy	0.78 (< 0.001)	1					
Professional self‐concept	0.65 (< 0.001)	0.63 (< 0.001)	1				
Gender role conflict	−0.17 (0.013)	−0.16 (0.030)	−0.01 (0.882)	1			
Clinical experience	−0.03 (0.632)	0.02 (0.816)	0.06 (0.409)	0.03 (0.701)	1		
Social support	0.77 (< 0.001)	0.73 (< 0.001)	0.59 (< 0.001)	−0.26 (< 0.001)	−0.03 (0.728)	1	
Nursing work environment	0.70 (< 0.001)	0.65 (< 0.001)	0.65 (< 0.001)	−0.21 (0.003)	−0.18 (0.011)	0.68 (< 0.001)	1

### 3.5. Hierarchical Linear Regression Analysis for Professional Socialization

To identify individual, interpersonal, and organizational factors associated with professional socialization, we conducted hierarchical linear regression. Before conducting the regression analysis, we examined the standard assumptions of independence, normality, and homoscedasticity. Independence was assessed using the Durbin–Watson statistic; the value for the final model was 1.835, which is close to 2 and indicates little evidence of autocorrelation among the residuals. Normality of residuals was evaluated visually using normal Q–Q plots and formally using the Shapiro–Wilk test. The normal Q–Q plot showed that the residuals closely followed the reference line, and the Shapiro–Wilk test did not indicate a substantial deviation from normality (*W* = 0.99, *p* = 0.23), suggesting that the normality assumption was reasonably satisfied. Homoscedasticity was assessed using residual‐versus‐fitted plots, which revealed no discernible pattern, indicating that the assumption of constant variance was met. To assess multicollinearity among the independent variables, we computed the variance inflation factor (VIF) in the final model; VIF values exceeding 10 were considered indicative of collinearity. All observed VIF values ranged from 1.11 to 2.67, confirming the absence of multicollinearity.

Model 1 included self‐efficacy, professional self‐concept, gender role conflict, and clinical experience as individual factors. Self‐efficacy and professional self‐concept were positively associated with professional socialization, whereas gender role conflict and clinical experience were not. The explained variance (adjusted *R* ^2^) was 0.621. Model 2 added social support as an interpersonal factor, and it was found to have a positive association with professional socialization, raising the explained variance to 0.693 (Δ adjusted *R* ^2^ = 0.072, *p* < 0.001). Model 3 added an organizational factor, and the nursing work environment also had a significant positive association with professional socialization, increasing the explained variance to 0.705 (Δ adjusted *R* ^2^ = 0.012, *p* < 0.001). In the final model (Model 3), self‐efficacy (*β* = 0.30, *p* < 0.001), professional self‐concept (*β* = 0.13, *p* = 0.028), social support (*β* = 0.37, *p* < 0.001), and nursing work environment (*β* = 0.18, *p* = 0.004) were positively associated with professional socialization, whereas gender role conflict (*β* = 0.05, *p* = 0.281) and clinical experience (*β* = −0.01, *p* = 0.894) were not (Table 5).

**TABLE 5:** Hierarchical linear regression analyses for professional socialization (*N* = 194).

Variable	Model 1				Model 2				Model 3			
	B	SE	*β*	*t*	B	SE	*β*	*t*	B	SE	*β*	*t*
Self‐efficacy	0.84	0.09	0.57	9.78^∗∗∗^	0.49	0.09	0.33	5.36^∗∗∗^	0.44	0.09	0.30	4.77^∗∗∗^
Professional self‐concept	0.64	0.12	0.30	5.17^∗∗∗^	0.41	0.12	0.19	3.59^∗∗∗^	0.27	0.12	0.13	2.21^∗^
Gender role conflict	−0.03	0.03	−0.04	−0.97	0.02	0.03	0.03	0.64	0.03	0.03	0.05	1.08
Clinical experience	−0.01	0.01	−0.06	−1.36	−0.01	0.01	−0.04	−1.04	−0.00	0.01	−0.01	−0.13
Social support					0.49	0.07	0.42	6.77^∗∗∗^	0.43	0.07	0.37	5.75^∗∗∗^
Nursing work environment									0.23	0.08	0.18	2.91^∗∗^

Adjusted *R* ^2^	0.621				0.693				0.705			
Δ adjusted *R* ^2^					0.072				0.012			
F (df1, df2)	79.96 (4, 189)				88.3 (5, 188)				78.0 (6, 187)			
Δ F (*p*)					45.84 (< 0.001)				8.49 (0.004)			

*Note:* Model 1 included self‐efficacy, professional self‐concept, gender role conflict, and clinical experience. In Model 2, social support was added. In Model 3, nursing work environment was added.

^∗^ *p* < 0.05.

^∗∗^ *p* < 0.01.

^∗∗∗^ *p* < 0.001.

## 4. Discussion

This study provides empirical evidence that professional socialization among male nurses can be understood within Bronfenbrenner’s ecological systems framework. Overall, professional socialization is shaped not only by individual resources (self‐efficacy and professional self‐concept) but also by interpersonal and organizational contexts, including social support and the nursing work environment. This perspective highlights the importance of nursing management strategies that integrate individual‐, interpersonal‐, and organizational‐level approaches, while also acknowledging societal influences such as public narratives and policy environments.

The level of professional socialization found in this study was moderately high, consistent with or slightly exceeding scores reported in a previous study of hospital nurses using the same scale [17]. This finding suggests that male nurses can attain levels of professional socialization comparable to those of female nurses, despite working in a female‐dominated profession. Notably, the perception of respect and recognition remained the lowest‐rated aspect in both our study and the prior study [17], which suggests that nurses continue to feel less recognized—a trend possibly reflecting ongoing societal undervaluation of nursing roles. Although this interpretation is based on descriptive ranking and differences between subfactor means were not formally tested, this common pattern across studies indicates a need for management interventions focusing on recognition and respect in the workplace. In addition to unit‐level recognition practices, nurse leaders should pursue long‐term, strategic, societal‐level approaches—working with institutional communications and engaging local and national media, including social media—to improve public understanding of the scope and complexity of nursing and strengthen the profession’s public image [25].

In our study, clinical experience was not significantly associated with professional socialization. This finding adds to the mixed evidence in previous research, where some studies have reported positive associations between clinical experience and professional socialization [17, 26], whereas others have found no relationship or even higher socialization scores among less experienced nurses [24, 27]. Although clinical experience is often assumed to contribute to the progressive refinement of clinical judgment and practical skills [13], this does not necessarily imply that professional socialization increases linearly with years of practice. A recent grounded theory study with nurses showed that professional socialization is fostered through processes such as developing a sense of belonging, enhancing communication, overcoming self‐doubt, and engaging in ongoing learning, which are shaped by supportive relationships, constructive feedback, and opportunities for workplace learning [28]. This suggests that professional socialization—understood as the internalization of the knowledge, skills, attitudes, beliefs, values, norms, and ethical standards expected of professionals [8]—is likely to depend more on the developmental quality of clinical experience than on its duration alone. Future longitudinal research should therefore disentangle the effects of the quantity and quality of clinical experience on the trajectory of professional socialization.

The positive associations of self‐efficacy and professional self‐concept with professional socialization underscore the role of individual resources in the socialization process among male nurses. Nurses’ professional self‐concept, built on self‐acceptance and self‐worth, fuels self‐efficacy [29]. Self‐efficacy, in turn, motivates employees to perform their duties effectively, positively impacting their professional self‐concept [30]. This mutually reinforcing relationship between self‐efficacy and professional self‐concept underscores the importance of fostering successful professional socialization. To enhance and sustain nurses’ self‐efficacy and professional self‐concept, a combination of individual‐level strategies (e.g., setting specific goals, self‐monitoring, and increasing leadership) and organizational‐level initiatives (e.g., peer support groups and counseling programs addressing work‐related challenges) should be implemented [31].

The mean gender role conflict level among our participants was comparable to those reported in previous studies of Korean male nurses [32, 33], but clearly lower than that observed in a large sample of adult men from the general population [21]. This pattern suggests that male nurses experience less gender role conflict than men in general, which may partly reflect the fact that men who choose nursing have already engaged in considerable reflection about gender norms and potential role conflicts when deciding to enter a female‐dominated profession [32, 33].

Gender role conflict showed a significant bivariate correlation with professional socialization; however, this association attenuated to nonsignificance after adjusting for self‐efficacy and professional self‐concept in multivariable models. A possible explanation is that the effect of gender role conflict on professional socialization may operate indirectly by reducing self‐efficacy and weakening professional self‐concept—both of which are critical for role integration and the internalization of professional norms, key aspects of professional socialization [34, 35]. Accordingly, the present results do not imply that gender role conflict is irrelevant; rather, its apparent association with professional socialization is indirect, operating via individual psychological factors such as self‐efficacy and professional self‐concept. In the Korean context, prejudices against male nurses remain prevalent [5], and stereotypes questioning their masculinity or role suitability persist [4]. Such sociocultural pressures may intensify gender role conflict and, in turn, indirectly hinder their professional socialization by undermining these crucial psychological mediators. From a nursing management perspective, gender role conflict should be framed as a mitigable barrier that nursing organizations can attenuate through organizational and societal‐level levers [25]. Leaders can pair visible internal recognition with public‐facing communications and media engagement to enhance the legitimacy and visibility of male nurses and counter gendered stereotypes; these actions may indirectly support professional socialization by strengthening self‐efficacy and professional self‐concept [25, 36]. At the system level, collaboration with professional bodies and schools to establish antidiscrimination/harassment standards, reporting protocols, and gender‐sensitivity training, alongside organizational policies that safeguard preceptorship support and capacity, can address constraints that are difficult to resolve at the unit level [37].

Social support served as an essential interpersonal context that facilitates professional socialization among male nurses. Previous studies have highlighted that being accepted as a nurse by significant individuals such as family members or colleagues has a substantial impact on socialization [7, 12]. However, male nurses often face challenges in building relationships and adapting to their departments because of negative perceptions of female nurses, such as the stereotype that male nurses are not suitable for the nursing profession because of their lack of attentiveness and meticulousness [38]. This suggests the need for raising awareness among female colleagues to see male nurses without gender‐based preconceptions and biases. Furthermore, male nurses have reported feeling a lack of support due to the scarcity of male nurse role models [7]. Role models not only provide guidance and inspiration but also serve as benchmarks for professional and personal growth [4]. To address this, hospitals need to support networks that allow male nurses to build relationships with each other and implement programs in which male nurses who serve as positive role models also act as mentors. Furthermore, encouraging the visibility of male nurse managers in leadership roles may reinforce the message that male nurses can make meaningful contributions to the profession and healthcare organizations.

From an ecological systems perspective, the nursing work environment represents a key organizational context that can enable or constrain professional socialization. This aligns with findings from a previous qualitative study on male nurses [7], which indicated that unfavorable work environment, such as an excessive workload, can hinder professional socialization. In Korea, due to the shortage of nurses, senior nurses generally train new nurses while performing their own duties [39]. Consequently, new nurses may not receive sufficient time or high‐quality training and are frequently assigned to nursing duties before becoming accustomed to their tasks [39]. Furthermore, the average nurse‐to‐patient ratio per shift in South Korean general hospitals is 16.3 [40], compared to 5.3 in the United States and 5.4 to 13.0 in European countries [41], indicating a particularly heavy workload for Korean nurses. To address this issue and foster a more supportive environment for professional socialization, healthcare organizations should strive to maintain adequate nurse staffing levels.

### 4.1. Limitations of the Study

Our study has several limitations. First, because of the cross‐sectional design and the use of single‐source self‐report measures collected at the same time point, we cannot make causal inferences, and there is a possibility of common method bias that may inflate or deflate associations among variables. Future studies should use prospective designs and incorporate design‐stage remedies (e.g., separating measurements across time or sources) and analytic checks (e.g., a common latent factor approach) to minimize and assess method bias [42]. Second, although the gender role conflict tool used in this study was applied to male nurses [32, 33], it assesses gender role conflict in general situations. Therefore, there are limitations in measuring the specific gender role conflicts experienced by male nurses in their working environments. Finally, our findings may have limited generalizability because the sample was restricted to hospital nurses in Korea and recruitment relied on a nonprobability, hospital‐mediated online process. This approach may have introduced gatekeeping and differential exposure to the survey across institutions, increasing the likelihood of selection and nonresponse biases and potentially overrepresenting more engaged participants or supportive workplaces. Although regional quotas enhanced geographic coverage, they do not ensure representativeness of the national male nurse population; therefore, estimates should not be interpreted as nationally representative. Future research should use probability‐based sampling across diverse healthcare settings and countries, and—where feasible—longitudinal designs to evaluate generalizability and causal pathways.

### 4.2. Implication for Nursing Management

The findings of this study offer actionable insights for nurse managers and healthcare providers. First, fostering peer support networks and mentorship programs (particularly by showcasing successful male nurse role models) could enhance the professional socialization of male nurses by providing guidance and a sense of belonging. Second, nursing management should work to cultivate an inclusive workplace culture—for instance, by educating staff to dispel gender stereotypes—so that male nurses feel accepted and valued by their colleagues. Lastly, improving organizational conditions, such as maintaining adequate nurse staffing levels and fair workload distribution, is crucial; a supportive practice environment was shown to positively influence socialization. By implementing these strategies, healthcare organizations can facilitate better integration of male nurses into the team and potentially improve their professional socialization. In addition, nurse managers can extend their influence beyond the organization by engaging in media communication and policy advocacy (e.g., through professional associations and workforce standards) to improve public representations of male nurses and reduce gender‐based stigma in nursing.

## 5. Conclusions

This study is one of the first to quantitatively delineate how personal factors and contextual conditions jointly shape professional socialization among Korean male nurses, within the framework of Bronfenbrenner’s ecological systems theory. This study identified key factors associated with professional socialization among Korean male nurses. The findings highlight that enhancing male nurses’ professional socialization requires coordinated efforts across personal, interpersonal, organizational, and societal levels. At the personal level, male nurses can strengthen their socialization by cultivating high self‐efficacy and a positive professional self‐concept. Interpersonally, fellow nurses should work toward reducing gender‐based biases and accepting male nurses as equal colleagues. Organizationally, healthcare institutions should provide financial and administrative support for improving working conditions, such as building peer support networks, fostering male nurse role models, and maintaining adequate nursing staff levels. Finally, at the societal level, public campaigns and educational efforts are needed to promote the professional image of male nurses and challenge persistent stereotypes. These results offer data‐driven guidance for developing practical programs to support male nurses’ integration into the profession and improve retention through enhanced professional socialization.

## Funding

This study was supported by the Health Fellowship Foundation and Mi Ja Kim Global Leadership Research Fellowship funded by Yonsei University College of Nursing.

## Disclosure

This study is based on a part of the first author’s doctoral dissertation.

## Conflicts of Interest

The authors declare no conflicts of interest.

## Data Availability

The datasets are not publicly available due to ethical restrictions and the absence of participants’ consent for data sharing.
